# P-2149. The Spectrum of Non-Valvular Cardiac Aspergillosis: What We Know So Far

**DOI:** 10.1093/ofid/ofae631.2303

**Published:** 2025-01-29

**Authors:** Zaid Al khouri, Anood Alqura’an, Ruhul Munshi, Mohammad Alam, Alexandre Malek

**Affiliations:** Louisiana State University Health Sciences Center Shreveport, Shreveport, Louisiana; LSU HEALTH SHREVEPORT, shreveport, Louisiana; LSU Health Shreveport, Shreveport, Louisiana; Louisiana State University Health Sciences Center, Shreveport, LA, USA, shreveport, Louisiana; Louisiana State University Health Sciences Center Shreveport, Shreveport, Louisiana

## Abstract

**Background:**

Invasive aspergillosis is a life-threatening infection that affects immunocompromised hosts. Cardiac involvement is rare, but it is associated with a high-mortality rate. Most cases reported infective endocarditis secondary to Aspergillus. However, there is limited data about non-valvular cardiac aspergillosis (NVCA). We performed a systematic review and added one case seen at our institution to highlight more on NVCA.

PRISMA Flow Diagram NVCA

**Figure:**
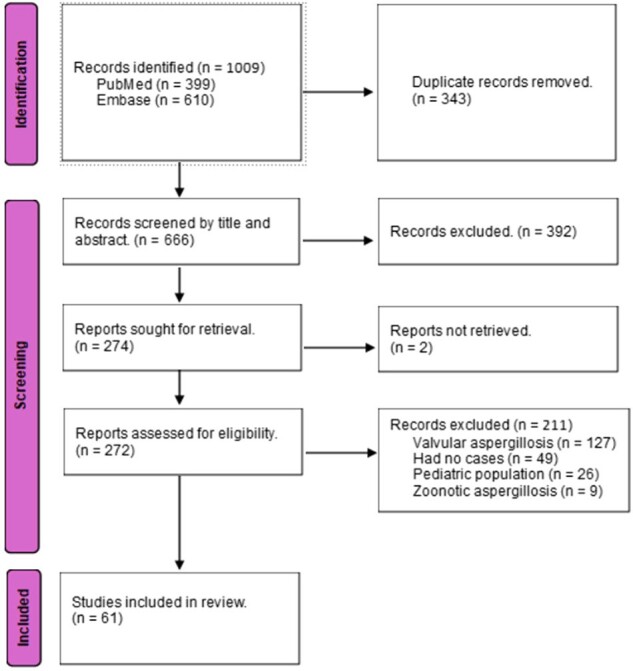

**Methods:**

Three reviewers searched PUBMED and EMBASE for all adult cases diagnosed with NVCA between 1950 and 2024 and assessed studies for eligibility (Fig 1). We added our case of 48 yo female with only congenital kyphoscoliosis diagnosed with isolated NVCA and was successfully treated with 6-month course of Voriconazole.

**Results:**

We found 67 cases including ours. A 36% were females and 64% males with a mean age of 44.6 y (range, 18-79). 30% of patients did have underlying hematologic malignancy and in 15% there were no comorbidities identified. Diagnosis was made by cultures and histopathology either from the pericardial fluid, cardiac masses, or from the cardiac layers, and it was antemortem only in 39%. It was identified in the pericardium alone in 40%. In 51% of cases, the Aspergillus species were not identified, while the remaining were secondary to A. fumigatus. TTE was done in 60% and showed pericardial effusion or tamponade in 31.5%. A 67% had lung involvement and 52% had invasive aspergillosis. In terms of outcomes, 19% remained alive, and those Pts received anti-fungal therapy including voriconazole in 69% & amphotericin in 46% +/- intervention in 61.5%, while 75% expired including 38% who did not receive management, but the remaining did mostly use amphotericin in 30%.

**Conclusion:**

NVCA is rare but highly fatal. Clinicians should keep a high-level of suspicion of this clinical entity, primarily in patients with liquid tumors and pericardial effusion. A combination of antifungal therapy, especially voriconazole along with minimally invasive or surgical intervention showed success in most cases.

**Disclosures:**

All Authors: No reported disclosures

